# A modified regimen of extracorporeal cardiac shock wave therapy for treatment of coronary artery disease

**DOI:** 10.1186/1476-7120-10-35

**Published:** 2012-08-17

**Authors:** Yu Wang, Tao Guo, Tie-kun Ma, Hong-yan Cai, Si-ming Tao, Yun-zhu Peng, Ping Yang, Ming-qing Chen, Yun Gu

**Affiliations:** 1 Department of Cardiology, 1st Hospital of Kunming Medical University, Kunming, Yunnan, PRC; 2 Department of Nuclear Medicine, 1st Hospital of Kunming Medical University, Kunming, Yunnan, PRC; 3 Department of Cardiology, 2nd People’s Hospital of Yunnan Province, Kunming, Yunnan, PRC; 4 Department of Cardiology, 1st People’s Hospital of Kunming, Yunnan, PRC; 5 President of 1st Hospital of Kunming Medical University, Kunming, Yunnan, PRC; 6 Department of Cardiology, Cardiovascular Center, 1st Hospital of Kunming Medical University, No.259, Xichang Road, Kunming, Yunnan, 650032, PRC

**Keywords:** Coronary artery disease, Angina pectoris, Myocardial ischemia, Cardiac shock wave therapy

## Abstract

**Background:**

Cardiac shock wave therapy (CSWT) improves cardiac function in patients with severe coronary artery disease (CAD). We aimed to evaluate the clinical outcomes of a new CSWT treatment regimen.

**Methods:**

The 55 patients with severe CAD were randomly divided into 3 treatment groups. The control group (*n* = 14) received only medical therapy. In group A ( *n* = 20), CSWT was performed 3 times within 3 months. In group B ( *n* = 21), patients underwent 3 CSWT sessions/week, and 9 treatment sessions were completed within 1 month. Primary outcome measurement was 6-minute walk test (6MWT). Other measurements were also evaluated.

**Results:**

The 6MWT, CCS grading of angina, dosage of nitroglycerin, NYHA classification, and SAQ scores were improved in group A and B compared to control group.

**Conclusions:**

A CSWT protocol with 1 month treatment duration showed similar therapeutic efficacy compared to a protocol of 3 months duration.

****Clinical trial registry**:**

We have registered on ClinicalTrials.gov, the protocol ID is CSWT IN CHINA.

## Background

The most common treatments for coronary artery disease (CAD) are medications combined with percutaneous coronary intervention (PCI) and coronary artery bypass graft surgery (CABG). While PCI and CABG are not without risks, overall results are satisfactory in patients who are suitable candidates. However, the prognosis for patients with refractory angina who are not candidates for PCI or CABG is poor as maximal medical therapy is ineffective in a large portion of these patients [1,2].

Shock wave (SW) therapy has been used successfully to treat renal calculi (lithotripsy) and for a number of orthopedic disorders [3,4]. Recently, experiments have shown that SWs with energy output at approximately 10 % of what is used for lithotripsy can promote neovascularization of cardiac tissue [5,6]. Subsequent clinical studies have shown that cardiac shock wave therapy (CSWT) can significantly improve cardiac function in patients with severe CAD and refractory angina who are not candidates for PCI or CABG [7-15]. One study that treated 24 patients with ischemic heart failure and a left ventricular ejection fraction (LVEF) < 40 % with CSWT demonstrated that the treatment significantly improved the cardiac functions assayed [9].

Though the results of CSWT for treating ischemic heart disease are encouraging, current protocol demands a treatment duration of three months. A one month protocol has not previously been developed, but a shorter protocol would save time and cost, produce better compliance, and might be a more suitable protocol than a three month protocol in China. Thus, the purpose of this prospective study was to evaluate the clinical outcome of a 1 month treatment regime as compared to the standard 3 month treatment regimen as well as to compare this treatment regime with controls who received medication only.

## Materials and methods

### Patients

This study was approved by the Institutional Review Board and Ethics Committee of 1^st^ Hospital of Kumming Medical University, and all study subjects signed informed consent for participation in the study and all treatments performed. Subjects were patients who were hospitalized at Department of Cardiology of our hospital from December 2008 to December 2009. Patients were eligible to be included in the study if they met any of the following criteria: 1) Coronary angiography (CA) or multi-slice CT coronary angiography (CTCA) suggestive of moderate to severe coronary artery stenosis. 2) Demonstrated cardiac infarction and > 50 % stenosis after radioactive and sonographic examinations. 3) Chest tightness, onset of shortness of breath, and poor exercise tolerance after receiving formal drug treatment (with or without stent or bypass graft). 4) Hospitalized more than 2 times within 1 year due to the aforementioned problems. 5) CCS angina grading higher than grade II, and NYHA functional classification of I-III. 6) More than 1 month after acute myocardial infarction (AMI) and more than 2 weeks after PCI surgery. A history of PCI or CABG was not a contraindication for inclusion. All patients included in the study were “no option” patients and had coronary heart disease and an imaging examination that confirmed the presence of ischemic myocardium. The diagnosis and treatment in all the patients was in accordance with the related domestic and European guidelines developed in 2007 [16].

Patients were excluded from the study if they met any of the following criteria. 1) AMI or CABG within the 4 weeks prior to the study. 2) History of heart transplantation. 3) History of metal valve replacement surgery. 4) Intracardiac thrombus. 5) LVEF < 30 % and unstable hemodynamics. 6) Arrhythmia with a rate < 40 bpm or > 120 bpm. 7) Skin ulceration or infection in the treatment area. 8) Severe obstructive lung disease.

### Grouping

Initially, consecutive patients who met the inclusion criteria were randomly divided into the regimen A treatment group and the regimen B treatment group. After three months, the additional group, a control group, was added and subsequent patients were randomly added to the regimen A and B groups and the control group. Patients in the three groups were enrolled by a physician who was familiar with patients’ conditions, and that physician drew lots to divide the patients into the different groups. The experimental grouping was blinded to both the physicians who were responsible for treatment and follow-up and to the patients themselves. The majority of patients with coronary heart disease in this study had severe symptoms so that they had a strong desire to receive CSWT. Therefore, we pre-set a smaller sample size for the control group and the sample size for this group was limited to no more than 15 patients. The control group did not receive CSWT. The treatment protocol of group A followed the recommended protocol developed by Tohoku University of Japan with respect to the shockwave output and the number of shots delivered to each spot and the protocol developed by the University of Essen, Germany [10,11]. In group A, a sequence of 1 week treatment followed by three weeks rest was repeated 3 times. During each treatment week, CSWT was performed on days 1, 3 and 5, one session each day. The total duration of treatment was 3 months, thus patients received a total of 9 CSWT treatment sessions. In group B, a modified CSWT treatment schedule was adopted. Patients underwent 3 CSWT sessions/week, and the 9 treatment sessions were completed within 1 month. During follow-up, if the patient exhibited no observable lessening of myocardial ischemia, 1–4 treatment courses were repeated.

### Examinations before treatment

Dobutamine stress echocardiography (DSE). Patients underwent DSE after vascular lesions were identified by CA and/or CTCA. The use of β-blockers, calcium antagonists, and nitrates were stopped for at least 24 hours before testing. Continuous intravenous infusion of dobutamine (5–10 μg/kg/min) was administered, and if regional wall motion abnormalities (RWMA) could not be induced by low-dose dobutamine the dose was increased to 20–40 μg/kg/min. The wall motion score index (WMSI) was used to assess the state of motion.

**Figure 1:**
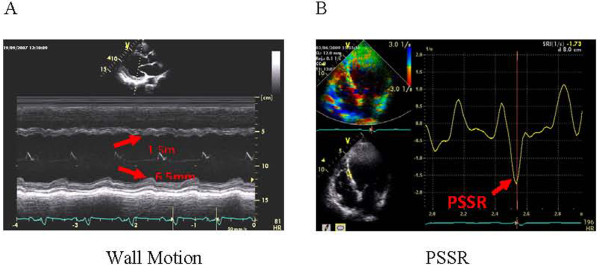
**Imaging methods.** ( **A**) Amplitude of regional myocardial motion was the range of motion measured using M-type echocardiography. ( **B**) PSSR was the measurement of the peak systolic strain rate using tissue Doppler imaging.

Amplitude of regional myocardial motion was the range of motion measured using M-type echocardiography (Figure 1A). This allows semiquantification of myocardial motion which can be displayed as a standard M mode image. Viable myocardial segments were defined as the 2 adjacent abnormal segments with improvement in contraction during drug administration (decrease ≥ 1 point) [17,18]

Tissue Doppler-based strain rate (SR) was used to measure peak systolic (PS) SR under resting and load conditions (Figure 1B) [19,20]. Strain rate measures the rate of deformation of a tissue segment and is measured in S^−1^. PSSR represents the maximal rate of deformation in systole. Strain is obtained by integrating strain rate over time and represents deformation of a tissue segment over time. Strain is expressed as the percent change from the original dimension. Systolic strain represents the magnitude of deformation between end diastole used as a reference point and end systole. One of authors of this paper carried out the PSSR measurement for all the patients during the entire follow-up period. However, that author did not know the treatment of each patient. Before this study, that author conducted a preliminary study on the feasibility and repeatability of PSSR measurement of all 16 segments of the left ventricle and the results showed no statistically significant difference (*P >* 0.05). Before CSWT treatment, radionuclide imaging and stress echocardiography were used to locate the ischemic segments in each patient. These segments might be the middle or the apical segment of interventricular septum, or the lateral basal segment. The PSSR was measured in the specific ischemic segments.

### Resting and dobutamine stress myocardial perfusion imaging (MPI)

In order to assess myocardial perfusion more accurately, identify the target for CSWT, and evaluate the therapeutic effects during follow-up, MPI was performed after vascular lesions were identified. The use of β-blockers, calcium antagonists, and nitrates were stopped at least 24 hours before testing. A two-day method was adopted for dobutamine load and resting technetium (TC-99 m) methoxy isobutyl isonitrile (^99m^Tc-MIBI) MPI. The patient was asked to consume a high-fat meal after injection of contrast medium on the first day. Intravenous continuous infusion of dobutamine (20–40 μg/kg/min) was performed 40 min after the contrast medium in the biliary tract was excreted and it was required that the target heart rate (220 - age) was reached. Resting scan was performed 40 min after injection of contrast medium on the second day. Target myocardium was located and quantified according to the 17-segment Myocardial Scoring and the 4-grade scoring system recommended by American Society of Nuclear Cardiology [21-23]. An increase of 1-point or more in MPI compared to baseline under both basic and loaded conditions was considered as the criterion for local myocardial blood flow improvement.

### Six-minute walk test (6MWT)

A 20-meter quiet and spacious corridor was designated, and the patients were asked to walk in the corridor at the maximum speed. If the patient experienced fatigue, dizziness, angina, or shortness of breath within 6 min, the test was stopped.

### CSWT treatment and control group

CSWT was performed with the MODULITH SLC SW therapy device (Storz Medical, Switzerland). In brief, after performing a 12-lead ECG, an ultrasound probe was used to locate the target myocardium based on the results of preoperative testing. A water bag was lowered until it touched the chest wall. Shock waves were applied to the ischemic areas, and were triggered by the R-wave of the ECG when the instrument was activated. The shock wave energy was increased up 0.09 mJ/mm^2^ if the patient did not experience discomfort such as chest pain. Point-to-point combination treatment (8 extra shock wave treatments around the ischemic area) with 200 pulses delivered to each point was given. Thus, there were 1800 shock waves administered for each infarcted segment.

The control group did not undergo CSWT. During the 12-month follow-up, periodic telephone inquiries, out-patient follow-up, and hospitalization were used to adjust the drugs and treat emergencies in addition to the regular 3-month, 6-month, and 12-month follow-ups.

In both A and B groups, if acute heart failure, unregulated blood pressure, and frequent chest tightness occurred, the treatment was supplemented by timely drug reinforcement and interventional treatment.

### Follow-up

Patients were followed-up at 3, 6, and 12 months after completion of treatment. Physicians who performed the follow-up examinations were not aware as to which treatment the patients received or if they were in the control group. Follow-up examinations included clinical assessment using the CCS grading of angina, NYHA functional classification, Seattle Angina Questionnaire (SAQ) [24], 6MWT, and recording the nitroglycerin dosage (times/week). Morphological assessment included measurements of the left ventricular end-diastolic diameter (LVDd) (two-dimensional long axis view of the left ventricle), left ventricular end-diastolic volume (EDV) (Simpson method), left ventricular end-systolic volume (ESV) (Simpson method), LVEF (Simpson method), regional wall motion under resting and load conditions (M-type measurement), PSSR under resting and load conditions (quantitative analysis using tissue velocity imaging), MPI score under resting and load conditions (semi-quantitative analysis using bulls-eye map).

### Statistical analysis

Data were summarized as mean ± SD for continuous data with a normal distribution, median with interquartile range (IQR: Q1, Q3) for data not normally distributed, and as number (%) for categorical data. Differences among groups were compared using one-way ANOVA with a post-hoc Bonferroni adjustment for continuous data or Kruskal-Wallis test for continuous data that were not normally distributed; Pearson chi-square test or Fisher's exact test was used for categorical data. ANCOVA was applied for comparing the effect between groups and within groups. Non-parametric methods, Kruskal-Wallis and Mann–Whitney U tests, were used for comparing differences among groups, and pair-wise comparison and Wilcoxon sign-rank test were used for comparing the differences within groups as data were not normally distributed. McNemar’s test was also applied for identifying the rate of subjects with improvement MPI within groups. The 6MWT was designated as the primary endpoint, and the b value of the 6MWT as estimated by the difference between baseline and the 12 month time point was 91.39 % for the control vs. group A, 70.15 % for the control vs. group B, and 12.17 % for group A vs. group B. All statistical assessments were two-tailed and a value of *P* < 0.05 was considered statistically significant. Statistical analyses were performed using SPSS 15.0 statistics software (SPSS Inc., Chicago, IL, USA).

## Results

A total of 55 patients who met the inclusion criteria were enrolled. Patient characteristics are summarized in Table 1. The 55 patients were randomly divided into one control group (*n* = 14) and two treatment groups: treatment group A ( *n* = 20) and group B ( *n* = 21). The mean age in the control group, group A, and group B was 67.9 ± 7.8 years, 62.7 ± 12.0 years, and 64.1 ± 9.8 years, respectively with no significant difference. The only significant difference between the groups was disease history ( *P* = 0.006). In the control group, all patients completed follow-up until 12 months. One patient experienced a recurrent AMI at the 3-month follow-up and underwent emergency PCI. In group A, 20 patients completed the 6-month follow-up and 19 completed the 12-month follow-up. One patient died from malignant arrhythmias which induced sudden cardiac arrest after the 6-month follow-up and 1 patient underwent PCI after the 6-month follow-up due to aggravated angina. In group B, 21 patients completed the 6-month follow-up and 10 patients completed the 12-month follow-up. One patient underwent PCI after the 6-month follow-up due to aggravated angina. Thus, 39 patients (19 in group A and 10 in group B) completed CSWT treatment and 12 months follow-up without heart failure, syncope, palpitations, breathing difficulty, bleeding, embolism, or shock.

**Table 1:** Subject characteristics

	**Control group (*n =*14)**	**Group A (*n =*20)**	**Group B (*n =*21)**	***P***
Age, years	67.9 ± 7.8	62.7 ± 12.0	64.1 ± 9.8	0.337
Sex, male (%)	12 (85.7)	18 (90)	17 (81)	0.882
BMI, kg/m^2^	24.0 ± 3.2	24.5 ± 2.8	23.4 ± 2.7	0.513
Disease history, years	3 (2, 5)	4 (2.0 , 7.8)	2 (1 , 2)	0.006*
Smokers	6 (42.9)	7 (35.0)	9 (42.9)	0.884
Underwent stenting	11 (78.6)	14 (70)	9 (42.9)	0.080
Comorbid conditions				
Essential hypertension	10 (71.4)	13 (65.0)	16 (76.2)	0.708
Diabetes mellitus	4 (28.6)	7 (35.0)	4 (19.0)	0.553
Hyperlipidemia	6 (42.9)	4 (20.0)	4 (19.0)	0.259
COPD	1 (7.1)	0 (0)	0 (0)	NA
Chronic renal failure	0 (0)	1 (5.0)	2 (9.5)	NA
Atrial fibrillation	0 (0)	1 (5.0)	0 (0)	NA
Ulcerative colitis	0 (0)	0 (0)	1 (4.8)	NA
Condition				0.383
Stable angina pectoris	2 (14.3)	0 (0)	0 (0)	
Old myocardial infarction	8 (57.1)	13 (65.0)	14 (66.7)	
Unstable angina pectoris	4 (28.6)	7 (35.0)	7 (33.3)	
Medical therapy				
Anti-platelet agents	9 (64.3)	11 (55.0)	14 (66.7)	0.774
Aspirin	12 (85.7)	17 (85.0)	19 (90.5)	0.883
Angiotensin-converting enzyme inhibitors	7 (50.0)	7 (35.0)	8 (38.1)	0.732
Angiotensin receptor blocker	1 (7.1)	4 (20.0)	4 (19.0)	0.659
β-blockers	13 (92.9)	18 (90.0)	18 (85.7)	0.872
Calcium channel blockers	4 (28.6)	6 (30.0)	11 (52.4)	0.281
Statin	13 (92.9)	18 (90.0)	17 (81.0)	0.675
Nitrates	4 (28.6)	8 (40.0)	9 (42.9)	0.727
Diuretics	2 (14.3)	3 (15.0)	3 (14.3)	1.000
Oral hypoglycemic agents	3 (21.4)	2 (10.0)	3 (14.3)	0.648
Insulin	2 (14.3)	2 (10.0)	1 (4.8)	0.623
Patients who experienced re-infarction	1 (7.1)	0 (0)	0 (0)	NA
Re-hospitalization				0.461
Once	4 (28.6)	2 (10)	2 (9.5)	
Twice	1 (7.1)	1 (5)	1 (4.8)	
Thrice	1 (7.1)	1 (5)	0 (0)	
Mortality	0 (0)	1 (5.0)	0 (0)	NA
Location of ischemic target area				
Ventricular septum	11 (78.6)	11 (55.0)	12 (57.1)	0.365
Anterior wall	2 (14.3)	4 (20)	4 (19)	1.000
Inferior wall	5 (35.7)	11 (55.0)	14 (66.7)	0.194
Posterior wall	1 (7.1)	3 (15.0)	3 (14.3)	0.771
Lateral wall	2 (14.2)	3 (15.0)	0 (0)	0.161
Number of shock wave treatment (9 times/per treatment course)	NA	2 (1, 2.8)	2 (1, 2.5)	0.904
Duration of treatment interval, month	NA	5 (4, 6.5)	4 (4, 5.5)	0.231

COPD: chronic obstructive pulmonary disease.

Data are presented as mean ± standard deviation, number (%), or median (IRQ) as described in the statistical analysis methods.

^*^P < 0.05, indicated significant difference was identified among control group, group A, and group B.

NA, not assessed.

There were no significant differences in myocardial enzymes or indicators of liver and kidney function between groups A and B before CSWT, or after the third and ninth treatment (*P* > 0.05). Premature ventricular contractions (PVCs) occurred in 4 cases in group A and 2 cases in group B during CSWT, but these did not affect blood pressure, heart rate, or oxygen saturation. The PVCs did not occur again during the following weeks of treatment. Three patients in group A experienced chest pain during the treatment of the ventricular septum and the apical segment, and it was relieved after the shock wave energy was reduced to 0.075-0.06 mJ/mm^2^. Those patients were then treated with the same lower energy, and chest pain did not occur again.

Table 2 shows the comparison of CCS grade, SAQ score, 6MWT, nitroglycerin dosage, and NYHA classification of the groups 0, 3, 6, and 12 months after treatment. Within-group comparisons (compared with month 0) revealed the mean CCS was significantly decreased in both groups A and B from month 3 to month 12. In the control group, mean CCS was only significantly decreased at month 6 as compared with month 0. The mean SAQ score at month 6 and month 12 in group A and at months 3, 6, and 12 in group B was significantly increased as compared with month 0. The mean 6MWT result at month 3, 6, and 12 in group A and at months 3 and 6 in group B was significantly increased as compared with month 0. We also performed a treadmill walk test for some, but not all, of the patients (Additional file 1: Table S1.) This test showed a significant increase in exercise tolerance in both CSWT groups, but not in the control group. The median NYHA classification score at month 3, 6, and 12 was significantly decreased as compared to month 0 in both group A and B (all, *P* < 0.05, Table 2).

**Table 2:** Comparison of CCS, SAQ, 6MWT, nitroglycerin dosage, and NYHA classification

	**Control group (14)**	**Group A (*n =*20)**	**Group B (*n =*21)**	***P***
6MWT, meters				
0 month	363.86 ± 150.92	344.25 ± 106.44	329.43 ± 134.71	0.617
3 month	322.07 ± 150.07	422.20 ± 77.30^§^	385.43 ± 78.62^§^	0.073
6 month	325.93 ± 157.32	434.25 ± 99.70^§^	405.33 ± 104.36^§^	0.296
12 month	348.43 ± 132.06	477.95 ± 105.34^a§^	452.00 ± 117.47^a^	0.020^*^
CCS grading of angina				
0 month	2 (2.0, 3.0)	2 (1, 2)	3 (2, 3)^b^	0.045^*^
3 month	2 (2.0, 2.3)	1 (1, 1)^a§^	2 (1, 2)^§^	<0.001^*^
6 month	2 (1.0, 2.3)^§^	1 (1, 1)^a§^	2 (1, 2)^§^	0.016^*^
12 month	2 (1.0, 3.0)	1 (1, 1)^a§^	1 (1, 2)^a§^	<0.001^*^
NYHA classification				
0 month	2 (1, 3)	1.5 (1, 3)	2 (1, 2.5)	0.822
3 month	1 (1, 3)	1 (1, 2)^§^	1 (1, 1)^§^	0.138
6 month	1 (1, 2.3)	1 (1, 1)^§^	1 (1, 1)^§^	0.081
12 month	1 (1, 2.3)	1 (1, 1)^§^	1 (1, 1)^a§^	0.018^*^
SAQ score				
0 month	63.21 ± 11.89	64.90 ± 11.72	67.71 ± 13.05	0.549
3 month	63.86 ± 13.27	69.50 ± 10.28	76.38 ± 13.20^a§^	0.015^*^
6 month	60.14 ± 12.82	75.00 ± 10.45^a§^	76.14 ± 12.28^a§^	<0.001^*^
12 month	59.21 ± 15.66	79.63 ± 9.87^a§^	82.70 ± 10.16^a§^	<0.001^*^
Nitroglycerin (times/week)				
0 month	1 (0, 4)	1 (0, 2)	2 (0, 3)	0.589
3 month	1 (0, 4)	0 (0, 1)	0 (0, 2)	0.151
6 month	0 (0, 2)	0 (0, 1)	0 (0, 1)	0.597
12 month^#^	1 (0, 3)	0 (0, 0)^a^	0 (0, 0)^a^	0.023^*^

Data were presented as mean ± standard deviation, number (%), or median (IRQ) as described in the statistical analysis methods.

^#^One patient in group A and 11 patients in group B had missing data of nitroglycerin dosage at month 12.

*P < 0.05, indicates significant difference among control group, group A, and group B.

^a,b^*P* < 0.0167 (0.05/3), indicates significant difference was identified when compared with ^a^control group and ^b^group A.

^§^*P* < 0.05, indicates significant difference was identified when compared with month 0 of the corresponding group.

**Figure 2:**
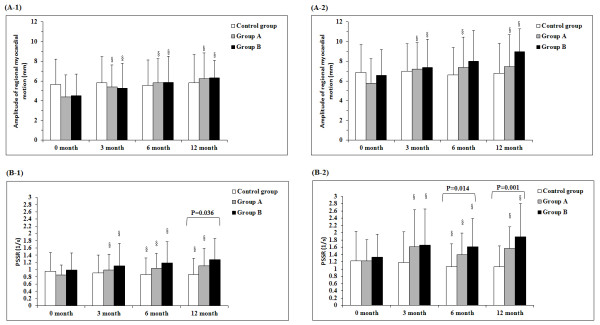
**Comparison of amplitude of regional myocardial motion at baseline (A-1) and after dobutamine loading (A-2), and PSSR score at baseline (B-1) and after dobutamine loading (B-2).** Data were presented as mean ± standard deviation as described in the statistical analysis methods. Twelve patients in group B had a missing record PSSR at baseline in 12 months. ^*^P < 0.05, indicated significant difference was identified among control group, group A, and group B. ^§^*P <* 0.05, indicated there was significant difference as comparing with time = 0 months for a given baseline or after load conditions in the corresponding group.

Figure 2 shows a comparison of the amplitude of regional myocardial motion and PSSR between- and within-groups. The baseline PSSR at month 12, PSSR after the load at month 6 and 12, and baseline MPI at month 0 were significantly different between the three groups. Within-group comparisons showed that for both group A and B, the amplitude of regional myocardial motion at month 3, 6, and 12 were all significantly higher than month 0 in both the baseline and the loaded conditions. For PSSR, the between-group comparisons showed significant differences at month 6 and 12 in both the baseline and loaded conditions.

**Figure 3:**
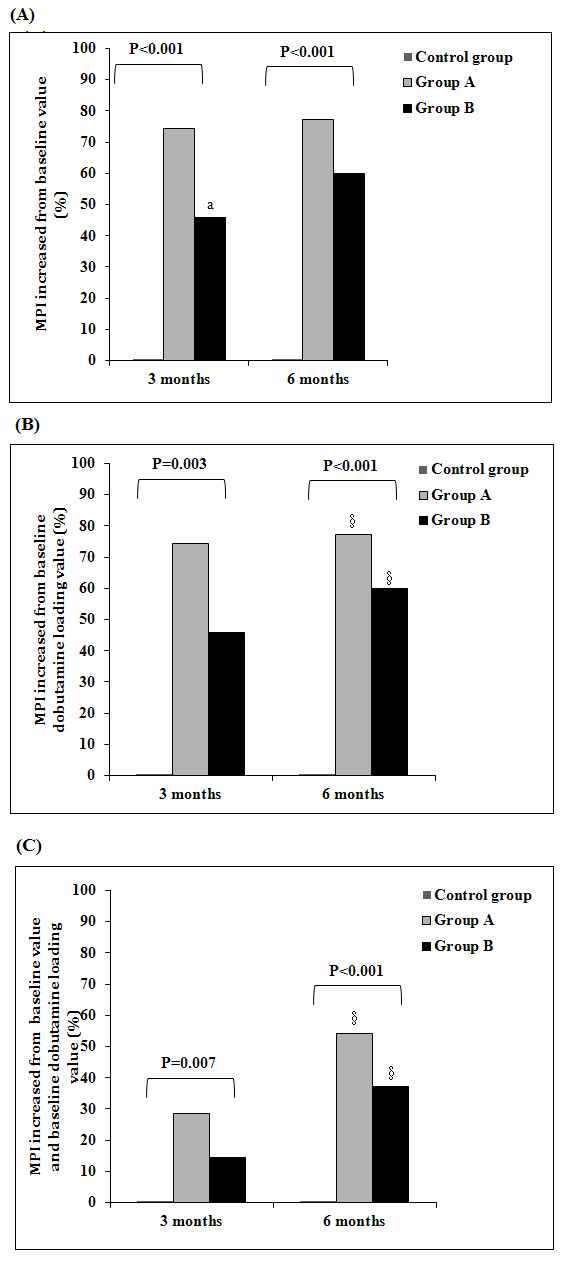
**Number of ischemic segments with increased baseline and post-dobutamine loading MPI scores.** Results were presented as bar chart as for the percentage of ( **A**) increased from baseline value at 3 month and 6 month for each group, ( **B**) increased from baseline dobutamine loading value at 3 month and 6 month for each group, and ( **C**) increased from baseline value and baseline dobutamine loading value at 3 month and 6 month for each group. * *P* < 0.05, indicates significant difference among groups through Fisher’s exact test. ^a^*P* < 0.0167 (0.05/3) indicates significant difference between group A and B through Chi-square test. ^§^*P* < 0.05 indicates significant difference within groups (0 to 6 months vs. 0 to 3 months) through McNemar’s test.

Results of MPI scores that measured the improvement of individual myocardial segments are shown in Figure 3. Successful treatment was defined as an increase of 1 point or more compared to month 0 in both baseline and loaded conditions. Although there is an increase in percentage of myocardia successfully treated in group A, the difference is not significant between group A and B.

## Discussion

In the current study, following 12 months of observation, the CSWT treatments using two different regimens both provided satisfactory results that improved myocardial function comparing to pretreatment (month 0) and to the control group. These results suggest a more frequent treatment regimen (one month) can also provide equivalent therapeutic efficacy compared to the regimen of less frequent CSWT treatment (three months).

PSSR, the technique used in this study, has higher temporal and spatial resolution than M type imaging [25,26]. Voight’s study showed that the peak systolic strain rate (PSSR) in the long-axis view decreased with the deterioration of the wall motion and that its sensitivity for determining myocardial ischemia was 86 % and its specificity 90 % in the dobutamine stress test [25] However, compared with the latest 2D-strain testing, PSSR is affected by the angle and can only measure the radial strain in the apical four-chamber and apical two-chamber view. It cannot measure the strain in the short-axis view. The present study selected PSSR to assess the local cardiac function because it was the earliest and most mature method available at the beginning of the study. Our research team members have used it for many years, therefore systematic errors can be reduced. Another reason for using PSSR is that we use GE VV7 instrument in our lab, and it has complete TDI and strain rate imaging software. The 2D-strain requires the SIMENS Sequia 512 instrument, and that instrument was not introduced until the late stage of this study. Therefore, in order to keep the consistency of results, we did not change the measurement indices.

Two prior studies were conducted by our research team. In the first, CCS grading of angina and dosage of nitrate esters were significantly reduced in 9 patients after 3 CSWT treatments and regional myocardial systolic function was improved significantly 1 month after treatment [12]. In the second, 25 patients had 9 CSWT treatments and two imaging methods were used, PSSR and MPI. In this study, the CCS grading of angina and dosage of nitrate esters, 6MWT, NYHA functional classification, and SAQ score were significantly improved and the PSSR after load and resting MPI were also significantly improved at the one month follow-up [13]. Other reports have also been encouraging. Fukumoto et al. [11] treated 9 patients with end-stage CAD who were not candidates for CABG or PCI, and at 12 months of follow-up reported that CSWT had significantly reduced nitroglycerine use (from 5.4 ± 2.5 to 0.3 ± 0.3 times/week), improved CCS functional class score (from 2.7 ± 0.2 to 1.8 ± 0.2), and improved myocardial perfusion as assessed by dipyridamole stress thallium scintigraphy (severity score, 25.2 ± 7.2 % improvement; extent score, 23.3 ± 9.0 % improvement). Khattab et al. [10] treated 10 patients with refractory AP who were CCS class III or IV despite maximal medical therapy with CSWT and reported that the mean CCS class decreased from 3.3 ± 0.5 at baseline to 1.0 ± 13 at follow-up and the mean summed stress score decreased from 8.3 ± 2.2 at baseline to 3.0 ± 3.1 at follow-up.

A shock wave propagates though water as a spike < 1 μs in duration with an amplitude up to 100 MPa, that is followed by a lower amplitude tensile portion lasting several microseconds [7]. Early studies with animal models of angina pectoris and AMI indicated that CSWT at approximately 10 % of the energy used for lithotripsy could improve left ventricular wall motion, LVEF, LV end-diastolic volume, and regional blood flow and number of capillaries in the border zone (MI) [7]. However, the molecular mechanism by which shock waves promote neovascularization and improvement of cardiac function has not been determined.

Shock waves have been reported to activate Ras, stimulate NO synthesis, produce anti-inflammatory effects by affecting the expression of chemokines and matrix metalloproteases, and upregulate VEFG and the VEGF receptor (Flt-1) [5,27-31]. How these effects lead to cardiac changes and improvement of cardiac function are not clear; however, it is possible that shock waves increase the incorporation of circulating endothelial progenitor cells (EPCs) by up-regulating the expression of stromal-derived factor 1 (SDF-1), which is necessary for the recruitment and incorporation of EPCs, in ischemic myocardium [7,32].

In our study, the one month treatment had the same efficacy as the 3 month treatment at the 12 month follow-up. These results are exciting, but the mechanism by which a shorter term, more frequent treatment produces the same effect as a longer term, less frequent treatment is still unclear. We speculate that the mechanism might be related to the cellular and molecular mechanisms of blood vessel formation [33-35]. In other words, when repeated shock wave stimulations are given within 1 month, the resulting succession of shear force effects will produce a waterfall phenomenon, and a large number of neovascular networks will form in a short period of time, ultimately promoting the establishment of collateral circulation in the ischemic area.

Limitations of the study are that we only used our second objective test of working capacity on a partial sample of the patient population. Also, because the patients in group B were admitted to the study later than the patients in group A, only 11 of the 21 patients in group B had been followed up at the time the paper was written.

## Conclusions

In summary, our results showed that CSWT can improve clinical symptoms and morphological and functional indices in patients with complex CAD. A CSWT treatment regimen of one month duration provided similar therapeutic efficacy compared to a regimen with three months duration. CSWT is proven effective and useful for patients excluded from CABG and PCI therapies and patients whose medication treatments are no longer effective.

## Abbreviations

6MWT: 6-minute walk test; CAD: Coronary artery disease; CSWT: Cardiac shock wave therapy; CCS: Canadian cardiology society; NYHA: New York heart association; SAQ: Seattle angina questionnaire; PSSR: Regional wall motion, peak systolic strain rate; MPI: Myocardial perfusion imaging.

## Competing interests

The authors declare no conflict of interest.

## Authors’ contributions

We declare that all the listed authors have participated actively in the study and all meet the requirements of the authorship. Dr. Tao Guo , Ming-Qing Chen, Yun Gu designed the study and wrote the protocol, Dr. Yu Wang, Hong-Yan Cai, Si-Ming Tao, Yun-Zhu Peng, Ping Yang performed research/study, Dr. Tie-Kun Ma, Hong-Yan Cai contributed important reagents, Dr. Yu Wang, Tao Guo managed the literature searches and analyses, Dr. Yu Wang,Si-Ming Tao undertook the statistical analysis, Dr. Yu Wang wrote the first draft of the manuscript. All authors read and approved the final manuscript.

## Funding source

None to declare.

## Supplementary Material

Additional file 1 Table S1. Comparison of treadmill exercise test for control, 3 month and 1 month CSWT patients. Click here for file
