# Supplementary material for: A modified regimen of extracorporeal cardiac shock wave therapy for treatment of coronary artery disease
Source: Cardiovasc Ultrasound. 2012 Aug 17;10:35. doi: 10.1186/1476-7120-10-35 (PMC3537548; doi:10.1186/1476-7120-10-35)
Supplement: Additional file 1 — Table S1. Comparison of treadmill exercise test for control, 3 month and 1 month CSWT patients. [file 1476-7120-10-35-S1.doc]

**Supplementary Table 1. Comparison of treadmill exercise test for control, 3 month and 1 month CSWT patients.**

|  | Control group (n=5) | Group A (n=7) | Group B (n=17) |
| --- | --- | --- | --- |
| Exercise time, seconds |  |  |  |
| 0 month | 232.6±50.71 | 325.71±58.02 | 354.59±36.20 |
| 3 month | 290.00±24.17 | 458.29±55.68^*^ | 462.59±33.90^*^ |
| 6 month | 377.60±61.27 | 583.14±46.57^*^ | 533.88±29.12^*^ |
| 12 month | 480.20±77.74 | 624.57±42.67^*^ | 595.63±41.66^*^ |
| Downward shift in the ST segment, mm |  |  |  |
| 0 month | 1.10±0.37 | 1.74±0.28 | 0.85±0.20 |
| 3 month | 0.74±0.27 | 1.46±0.29^*^ | 0.71±0.19^*^ |
| 6 month | 0.70±0.34 | 0.97±0.35^*^ | 0.61±0.19^*^ |
| 12 month | 0.50±0.39 | 0.86±0.30^*^ | 0.65±0.26^*^ |

Data were represented as mean±SD.

^*^P<0.05, indicates significant difference was identified when compared with month 0 of the corresponding group.
